# Xylosyltransferase-I serum activity is a promising biomarker for postmenopausal osteoporosis

**DOI:** 10.1038/s41598-026-66889-0

**Published:** 2026-08-20

**Authors:** Alina Witt, Massimo De Martinis, Matthias Kühle, Vanessa Schmidt, Ernesto Aitella, Cornelius Knabbe, Isabel Faust-Hinse

**Affiliations:** 1 Herz- und Diabeteszentrum Nordrhein-Westfalen, Institut für Laboratoriums- und Transfusionsmedizin, Universitätsklinik der Ruhr-Universität Bochum, 32545 Bad Oeynhausen, Germany; 2 Herz- und Diabeteszentrum Nordrhein-Westfalen, Medizinische Fakultät OWL (Universität Bielefeld), 32545 Bad Oeynhausen, Germany; 3 Department of Life, Health and Environmental Sciences, University of L’Aquila, L’Aquila, 67100 AQ Italy

**Keywords:** Osteoporosis, Xylosyltransferase-I, Postmenopausal women, Serum biomarker, Biomarkers, Diseases, Endocrinology, Medical research

## Abstract

Osteoporosis is characterized by reduced bone mineral density (BMD) and impaired bone microarchitecture, leading to increased fracture risk. Current bone turnover biomarkers reflect osteoclast and osteoblast activity but do not capture the status of bone extracellular matrix (ECM). Human xylosyltransferase (XT), which initiates glycosaminoglycan biosynthesis, is essential for ECM integrity and was recently implicated in skeletal disorders. To investigate its role in osteoporosis, serum activity of the two XT isoenzymes, XT-I and XT-II, were quantified in 40 postmenopausal women with osteoporosis and 40 age-matched healthy controls using a SPE-UPLC-MS/MS assay. Associations with BMD, fracture risk scores (FRAX) and markers of bone turnover were assessed using Pearson correlation analysis and principal component analysis (PCA). Serum XT-I activity was significantly reduced in postmenopausal osteoporosis patients, whereas XT-II activity remained unchanged. XT-I showed strong positive correlations with all BMD parameters and significant negative correlations with FRAX and bone turnover markers. PCA positioned XT-I activity alongside BMD parameters on the primary axis of disease-related variance, opposing bone resorption markers and fracture risk scores. These findings identify XT-I as a promising biomarker reflecting ECM remodeling in postmenopausal osteoporosis and support further investigation of its role in bone remodeling.

**Supplementary Information:**

The online version contains supplementary material available at 10.1038/s41598-026-66889-0.

## Introduction

Osteoporosis is a systemic skeletal disorder characterized by reduced bone mineral density (BMD) and impaired bone microarchitecture, leading to increased bone fragility and fracture risk^1^. The condition affects an estimated 200 million women worldwide and represents a major public health burden, particularly in aging populations^2,3^. Postmenopausal women are often disproportionately affected due to the sharp decline in estrogen levels, which disrupts the physiological balance of bone remodeling^4^. The latter depends on a tightly regulated interaction between osteoblast-driven formation and osteoclast-mediated resorption of bone tissue^5^. This balance is disrupted in favor of osteoclast activity. Not only decreased circulating levels of sex steroids but also multiple other mediators contribute to this pathological shift, including pro-inflammatory cytokines and receptor activator of nuclear factor-κB ligand (RANK-L) signaling^6^. The resulting excessive osteoclast differentiation and osteolysis lead to the progressive loss of trabecular and cortical bone that defines the osteoporotic phenotype^7^.

Glycosaminoglycans (GAGs) are sulfated polysaccharides that are major components of the extracellular matrix (ECM) of bone and cartilage and play critical roles in bone remodeling^8,9^. GAG chains are covalently attached to core proteins forming proteoglycans (PG). This reaction is initially catalyzed by the enzyme xylosyltransferase (XT)^10^. Two isoforms have been identified in humans: XT-I (encoded by *XYLT1*) and XT-II (encoded by *XYLT2*). Both isoforms catalyze the same rate-limiting step of PG biosynthesis, namely the transfer of xylose from UDP-xylose to specific serine residues of core proteins, thereby initiating the assembly of GAG chains such as chondroitin sulfate, heparan sulfate and dermatan sulfate. Despite their shared catalytic function, XT-I and XT-II exhibit distinct expression patterns and are associated with different disease phenotypes. The XT-II is ubiquitously expressed and represents the predominant isoform in most human tissues, whereas XT-I shows a more restricted, tissue-specific expression profile^11^. Current knowledge regarding the role of XT-I in bone biology is still limited. Experimental studies addressing *XYLT1* deficiency have predominantly been performed in mouse and zebrafish models, where the primary focus has been on endochondral ossification during embryonic skeletal development^12–14^. Accordingly, the expression and function of XT-I in adult bone have not yet been comprehensively characterized. However, recent studies demonstrated that *XYLT1* deficiency in human mesenchymal stem cells alters osteogenic differentiation and ECM composition, indicating that XT-I-mediated PG biosynthesis contributes to bone matrix homeostasis^15^. Together, these findings suggest that disturbances of XT-I activity may also influence bone remodeling in acquired skeletal diseases. The functional relevance is underscored by human genetic disorders affecting each isoform. Pathogenic *XYLT1* mutations lead to Desbuquois dysplasia type 2 (DBQD2; OMIM #608124), which presents with distinct skeletal abnormalities such as growth retardation, short long bones, and characteristic facial features^16^. By contrast, pathogenic *XYLT2* mutations cause spondylo-ocular syndrome (SOS; OMIM #605822), a rare autosomal recessive disorder characterized by severe osteoporosis, skeletal dysplasia, and multisystem involvement including ocular, cardiac, and hearing impairment^17^. Both DBQD2 and SOS are classified as GAG linkeropathies, which are associated with alterations in GAG biosynthesis, including reduced availability or altered sulfation of GAG chains, in which impaired linker formation leads to changes in ECM-composition and skeletal abnormalities^18^. Importantly, GAGs are key regulators of osteoclast differentiation and activity: reduced GAG availability has been associated with enhanced osteoclastogenesis and increased bone resorption in osteoclastic precursor cells^19^. At the molecular level, GAGs can influence the differentiation of osteoclasts in various ways. Heparan sulfate and chondroitin sulfate PG can directly inhibit RANKL–RANK interactions, thereby attenuating the downstream NF-κB signaling required for osteoclast precursor differentiation^20^. In addition, GAG chains regulate the bioavailability and signaling of bone-anabolic growth factors, including bone morphogenetic protein-2 and wingless-related integration site ligands, which are essential for osteoblast function and the maintenance of bone formation^21^. Disruption of this GAG-mediated regulatory network may therefore shift the balance of bone remodeling towards resorption and contribute to progressive bone loss. This suggests that impaired GAG biosynthesis is not only relevant in rare genetic disorders but may also play a role in the pathophysiology of osteoporosis. In this context, increased osteoclast activity may be accompanied by compensatory or dysregulated changes in ECM synthesis. Given its central role in initiating GAG biosynthesis, serum XT activity may therefore serve as a surrogate marker of ECM remodeling during osteoporotic bone turnover and may be detectable as a systemic biomarker of altered bone matrix dynamics. A distinctive feature of XT is its secretion into the extracellular space. While the initial xylosylation of PG occurs in the Golgi apparatus, the catalytic domain of XT can subsequently be cleaved by cysteine proteases and released together with the PG into the extracellular environment. Consistent with this, XT activity is measurable in human serum and thought to originate primarily from PG-synthesizing cells^18^. Consequently, circulating XT activity reflects the overall biosynthetic activity of GAG-producing cells and may serve as a systemic indicator of ECM remodeling. Notably, XT-II represents the predominant XT isoform in human serum under physiological conditions, whereas XT-I appears to be more dynamically regulated and released in response to pathological alterations in matrix turnover^22^. Consistent with this, elevated XT-I activity has been reported in fibrotic and inflammatory diseases, including systemic sclerosis and liver fibrosis, where it correlates with disease severity and ECM turnover^23–25^. Accordingly, alterations in circulating XT-I activity have been linked to disease activity, supporting its potential as a biomarker of matrix remodeling, while XT-II appears to serve primarily as a constitutive serum isoform with limited diagnostic relevance. Although associations between osteoclast differentiation and the availability of GAG have already been established, the role of XT activity in osteoporosis has not yet been addressed. Therefore, the aim of this study was to investigate the role of circulating XT-I and XT-II activity in postmenopausal women. We specifically aimed to (i) compare serum XT-I and XT-II activities between osteoporosis patients and healthy controls; (ii) determine their associations with BMD, fracture risk, and established bone turnover markers; and (iii) integrate XT activity into the global multivariate structure of osteoporosis-related parameters using correlation analysis and principal component analysis (PCA) to assess its position within the disease-related variance landscape, thereby evaluating its potential as a novel serum biomarker for osteoporosis and providing a rationale for future studies exploring XT as a possible therapeutic target.

## Results

**Fig. 1:**
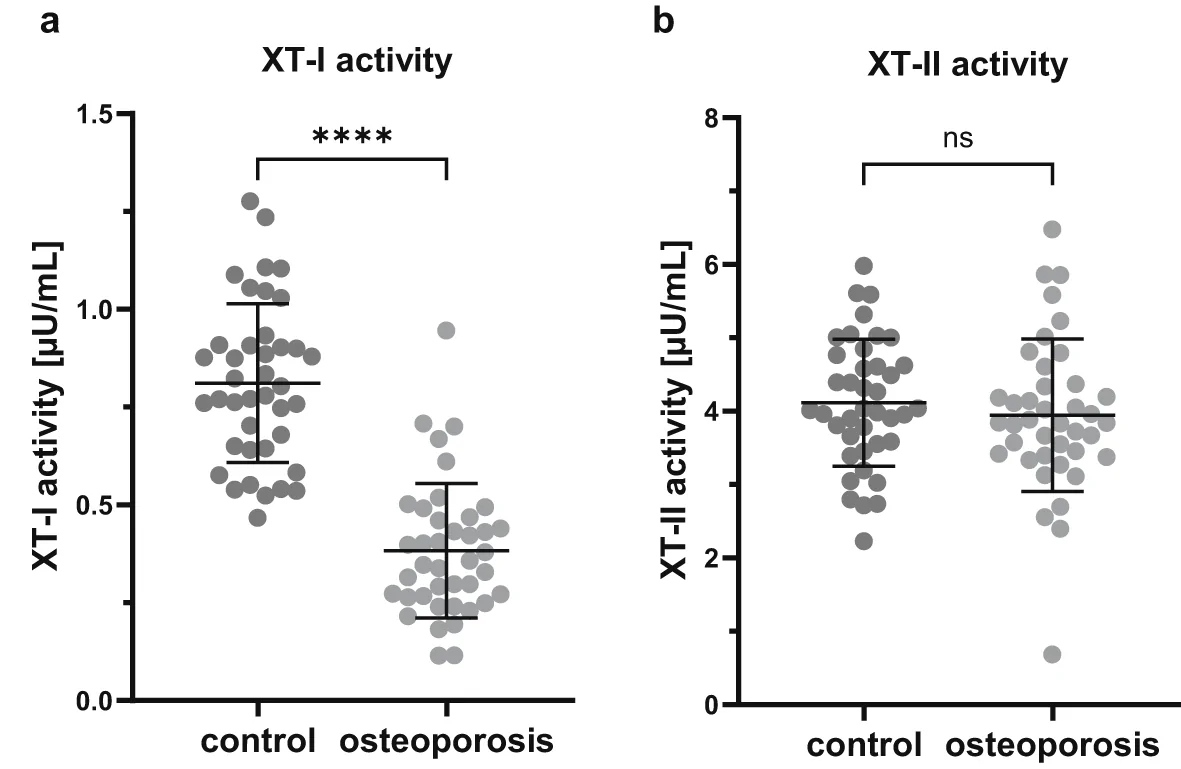
Serum XT-I and XT-II activity in osteoporosis patients and controls. Serum XT levels were determined using a SPE-UPLC-MS/MS-based assay for the selective, simultaneous quantification of XT-I and XT-II (n_technical_=2) and compared between postmenopausal osteoporosis patients (*n* = 40) and age-matched healthy controls (*n* = 40). With (**a**) Serum XT-I activity and (**b**) Serum XT-II activity Data are presented as mean ± SD. Statistical significance was assessed using the Mann-Whitney U test: ns (*p* ≥ 0.05; not significant); **** (*p* < 0.0001).

Mass spectrometric-determined serum XT-I activity was significantly lower in osteoporosis patients than in controls (approximately 0.46 ± 0.17 vs. 0.82 ± 0.22 µU/mL; *p* < 0.0001; Fig. 1a), indicating a strong and consistent effect across the patient population. By contrast, serum XT-II activity did not differ significantly between osteoporosis patients and controls (*p* ≥ 0.05 (ns); Fig. 1b). These results demonstrate that the reduction in circulating XT enzyme serum activity in postmenopausal osteoporosis is isoform-specific and confined to XT-I.

**Fig. 2:**
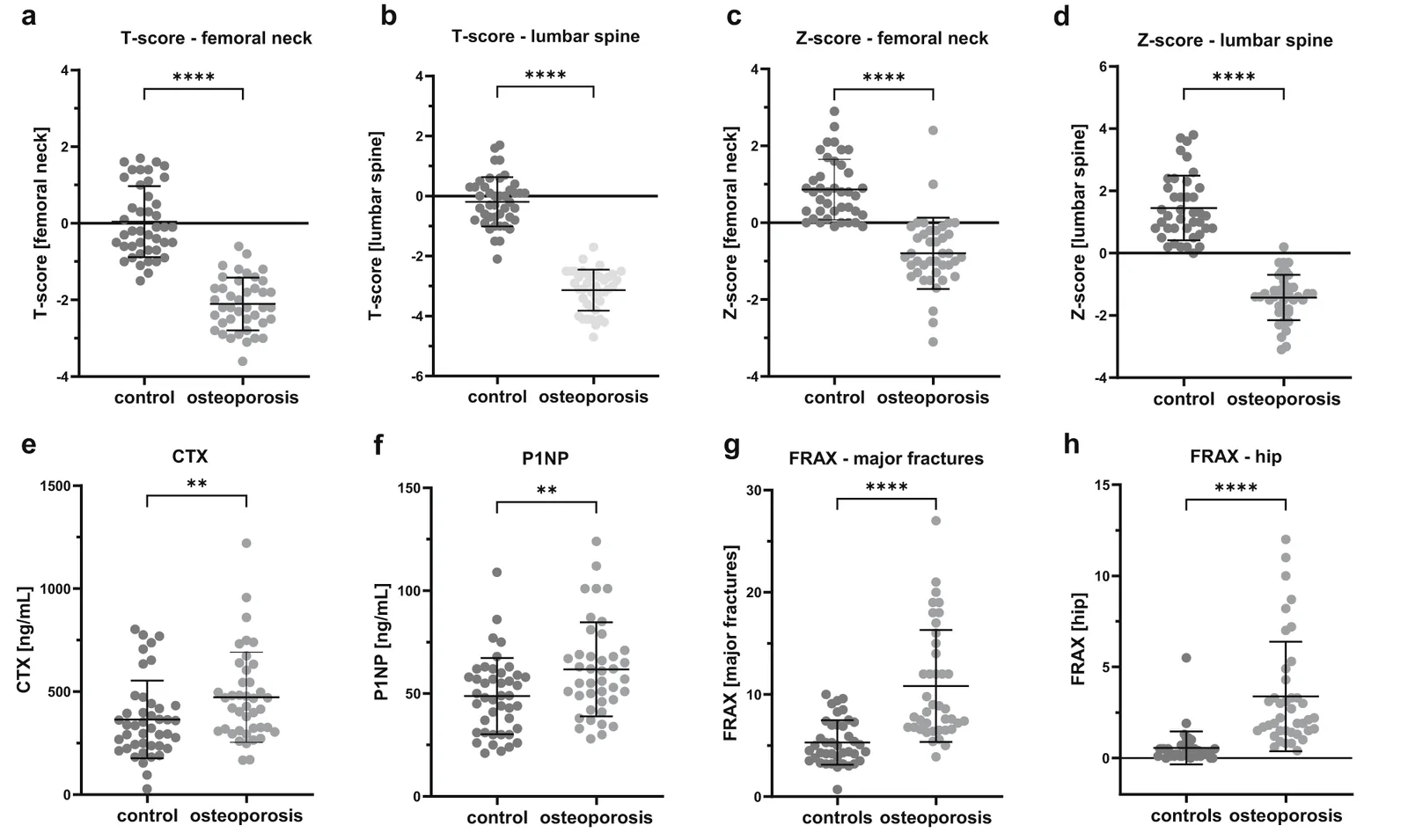
Validation of BMD, bone turnover parameters and FRAX in osteoporosis patients and controls. Serum levels and bone density parameters were compared between postmenopausal osteoporosis patients (*n* = 40) and age-matched healthy controls (*n* = 40). The BMD was measured at the femoral neck and lumbar spine using DXA. Routine laboratory parameters reflecting bone turnover were recorded. Ten-year fracture probabilities for major osteoporotic fractures and hip fractures were estimated for all participants using FRAX. With (**a**) T-score femoral neck, (**b**) T-score lumbar spine, (**c**) Z-score femoral neck and (**d**) Z-score lumbar spine, the bone resorption marker (**e**) CTX, the bone formation marker (**f**) P1NP, (**g**) FRAX major fractures and (**h**) FRAX hip. Data are presented as mean ± SD. Statistical significance was assessed using the Mann*-*Whitney U test: ** (*p* < 0.01); **** (*p* < 0.0001).

DXA-derived bone density parameters and established bone turnover markers were compared between osteoporosis patients and controls to confirm the validity of the study cohort. T-scores were significantly reduced in osteoporosis patients at both measurement sites: T-score femoral neck (controls: 0.05 ± 0.87 vs. osteoporosis: −2.20 ± 0.66; *p* < 0.0001, Fig. 2a) and T-score lumbar spine (− 0.15 ± 0.74 vs. −3.15 ± 0.69, *p* < 0.0001; Fig. 2b). Z-scores were similarly significantly lower in the osteoporosis group at the femoral neck (0.86 ± 0.79 vs. −0.92 ± 0.99; *p* < 0.0001, Fig. 2c) and lumbar spine (1.45 ± 0.38 vs. −1.43 ± 0.4; *p* < 0.0001, Fig. 2d). Consistent with enhanced bone remodeling activity in osteoporosis, the resorption marker CTX was significantly elevated in osteoporosis patients relative to controls (474.75 ± 16.58 vs. 360.77 ± 15.51 pg/mL; *p* < 0.01; Fig. 2e), as was the bone formation marker P1NP (61.88 ± 2.22 vs. 49.55 ± 2.42 ng/mL; *p* < 0.01; Fig. 2f). Furthermore, the fracture probabilities estimated by FRAX were increased in osteoporosis patients compared to controls (major fractures: 10.83 ± 5.44 vs. 5.32 ± 2.18, *p* < 0.0001; Fig. 2g; hip: 3.38 ± 3.02 vs. 0.55 ± 0.91, *p* < 0.0001; Fig. 2h). The significance and directionally expected differences in bone density and turnover parameters and FRAX confirm the suitability of the cohort for investigating novel osteoporosis-related evaluations.

**Fig. 3:**
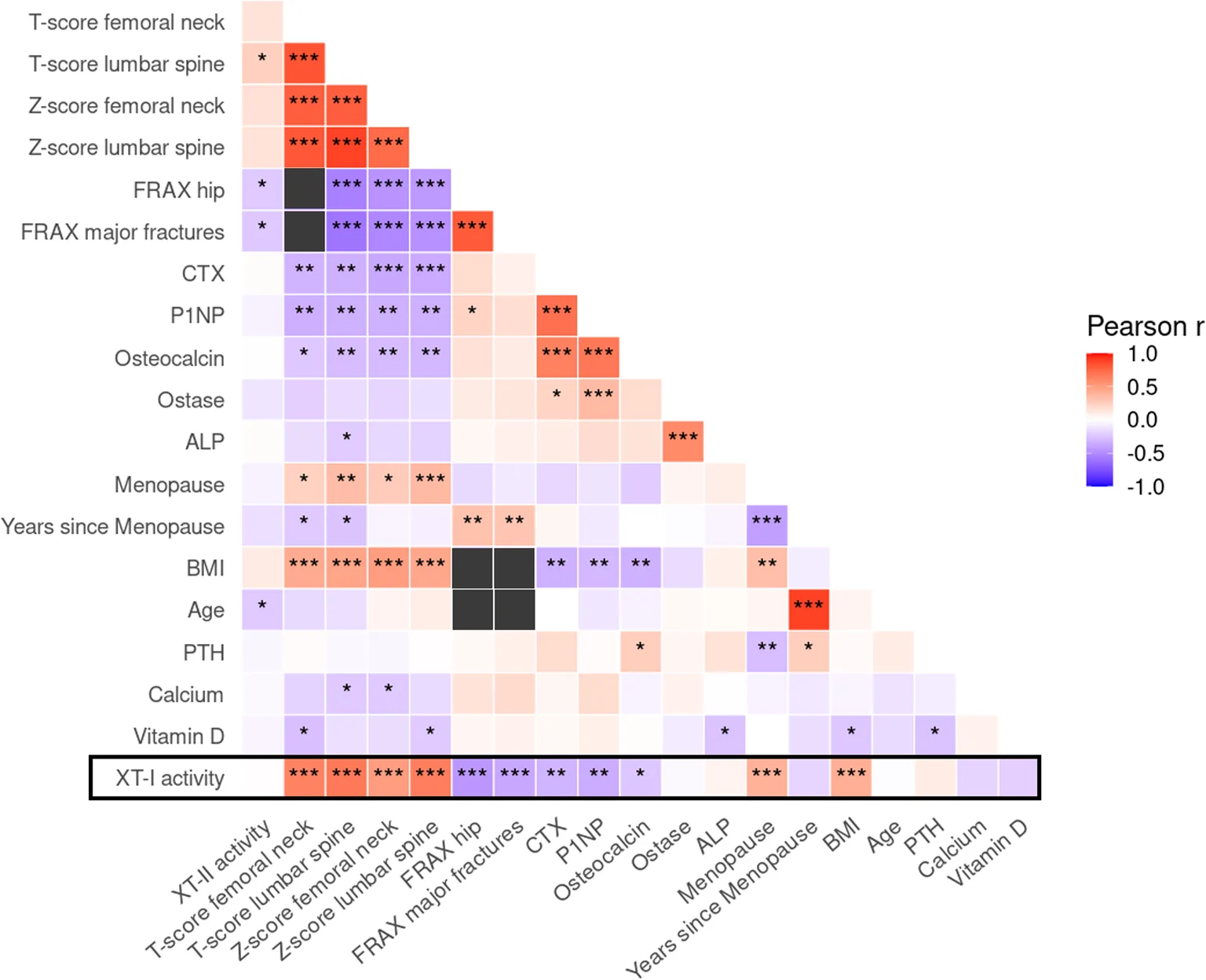
Pearson correlation matrix of all parameters measured in osteoporosis patients and controls. Serum levels were compared between postmenopausal osteoporosis patients (*n* = 40) and age-matched healthy controls (*n* = 40). Pairwise Pearson correlation coefficients between all clinical, anthropometric, and laboratory parameters are displayed as a color-coded heatmap. Red indicates positive correlation; blue indicates negative correlation; color intensity reflects the magnitude of the correlation coefficient (Pearson r scale: −1.0 to 1.0). Grey boxes indicate correlations that were intentionally omitted because Age, BMI and T-score femoral neck are incorporated into the FRAX algorithm and would therefore not represent independent associations. The XT-I activity row is highlighted by a black border. Statistically significant correlations are indicated: *( *p* < 0.05); ** (*p* < 0.01); *** (*p* < 0.001). The heatmap was generated by the authors using R (version 4.3.3; R Foundation for Statistical Computing, Vienna, Austria; available at: The R Project for Statistical Computing).

A pairwise Pearson correlation matrix was computed and visualized as a heatmap, with the XT-I activity column highlighted, to obtain a comprehensive overview of linear associations across all bone-related parameters measured (Fig. 3). The XT-I activity showed significant positive correlations with all four BMD parameters: T-score femoral neck (*p* < 0.001), T-score lumbar spine (*p* < 0.001), Z-score femoral neck (*p* < 0.001), and Z-score lumbar spine (*p* < 0.001). Further positive associations were observed with the BMI (*p* < 0.001) and menopause status (*p* < 0.001). Significant negative correlations were identified with fracture risk scores (FRAX hip and FRAX major fractures; both *p* < 0.001) and bone turnover markers CTX (*p* < 0.01), P1NP (*p* < 0.01), and osteocalcin (*p* < 0.05). The broader correlation structure revealed two parameter clusters: a bone density cluster (T-scores, Z-scores, XT-I, BMI, menopause) with strong positive intercorrelations and a bone turnover cluster (CTX, P1NP, osteocalcin) with significant internal intercorrelations and negative associations with the density cluster. The FRAX scores bridged both clusters, correlating negatively with bone density parameters and positively with turnover markers. Importantly, XT-II activity showed significant correlations with only a few parameters: age (*r* = -0.23, *p* < 0.05), FRAX scores (*r* = -0.24, *p* < 0.05), and T-score lumbar spine (*r* = 0.25, *p* < 0.05).

**Fig. 4:**
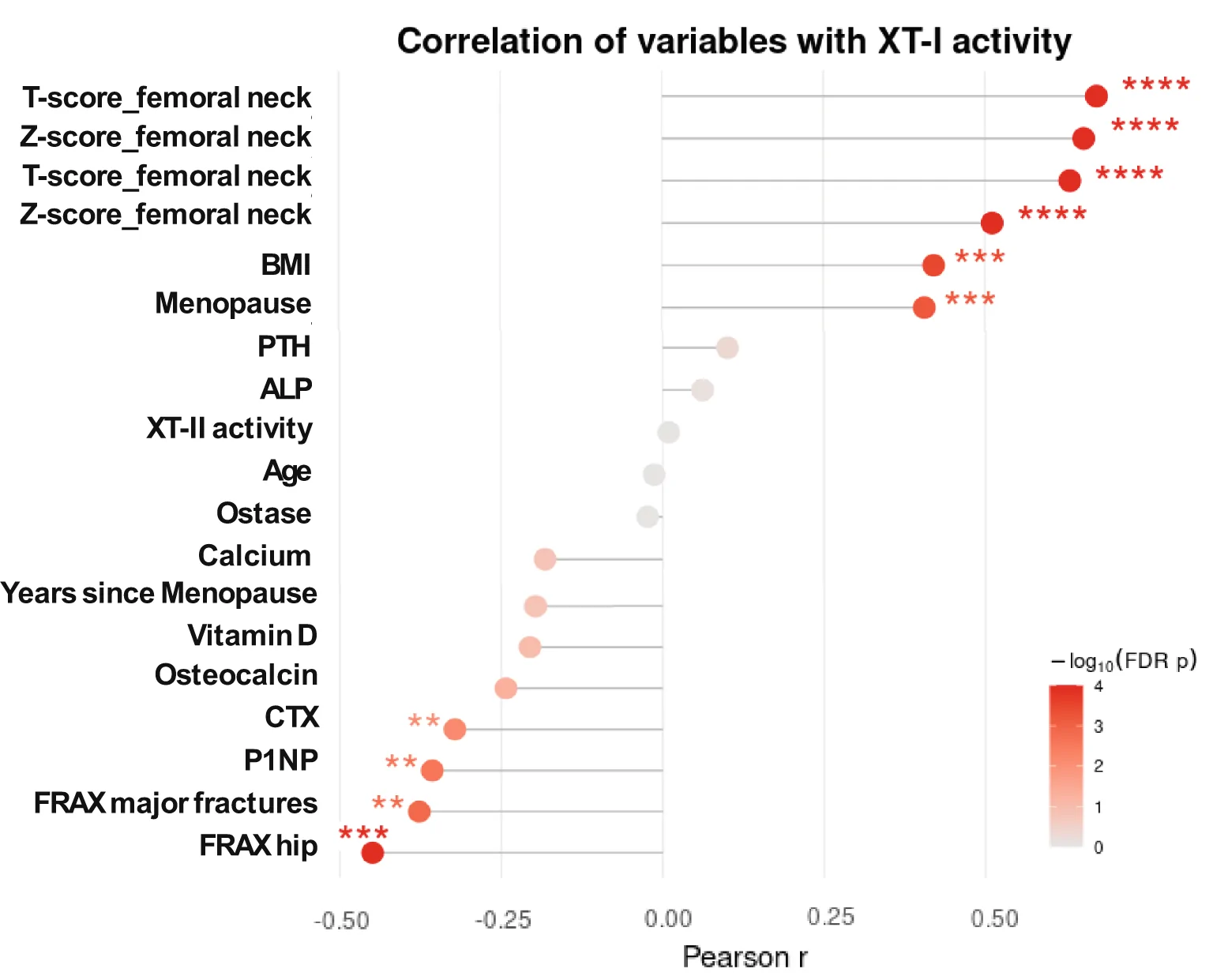
Pearson correlations of all parameters measured with serum XT-I activity in osteoporosis patients and controls. Serum levels were compared between postmenopausal osteoporosis patients (*n* = 40) and age-matched healthy controls (*n* = 40). Pearson correlation coefficients (r) between XT-I activity and all clinical, anthropometric, and laboratory parameters are displayed as a ranked dot plot. Positive values indicate a positive association with XT-I activity; negative values indicate an inverse association. Dot color reflects the FDR-adjusted significance level (− log₁₀(FDR p)); grey dots indicate non-significant correlations after FDR correction. Statistically significant correlations are indicated: ** (*p* < 0.01); *** (*p* < 0.001); **** (*p* < 0.0001).

A focused Pearson correlation analysis was performed against all bone-related parameters, ranked by effect size, and visualized with false discovery rate-adjusted significance, to characterize the associations of XT-I activity in detail (Fig. 4). The strongest positive correlations were observed with T-score lumbar spine (*r* = 0.67, *p* < 0.0001), Z-score lumbar spine (*r* = 0.65, *p* < 0.0001), T-score femoral neck (*r* = 0.63, *p* < 0.0001), and Z-score femoral neck (*r* = 0.51, *p* < 0.0001), confirming a consistent and quantitatively meaningful association between XT-I activity and BMD at both skeletal sites. Moderate positive correlations were additionally observed with the BMI (*r* = 0.42, *p* < 0.001) and menopause status (*r* = 0.41, *p* < 0.001). The strongest negative correlation was with FRAX hip (*r* = − 0.45, *p* < 0.001), followed by FRAX major fractures (*r* = − 0.38, *p* < 0.01), P1NP (*r* = − 0.36, *p* < 0.01), and CTX (*r* = − 0.32, *p* < 0.01). A weak negative trend was noted for osteocalcin. Age, XT-II activity, ostase, ALP, PTH, calcium, Years since Menopause, and vitamin D did not reach FDR-corrected significance, indicating that the associations of XT-I are specific to BMD and bone remodeling activity rather than to general metabolic or endocrine parameters.

**Fig. 5:**
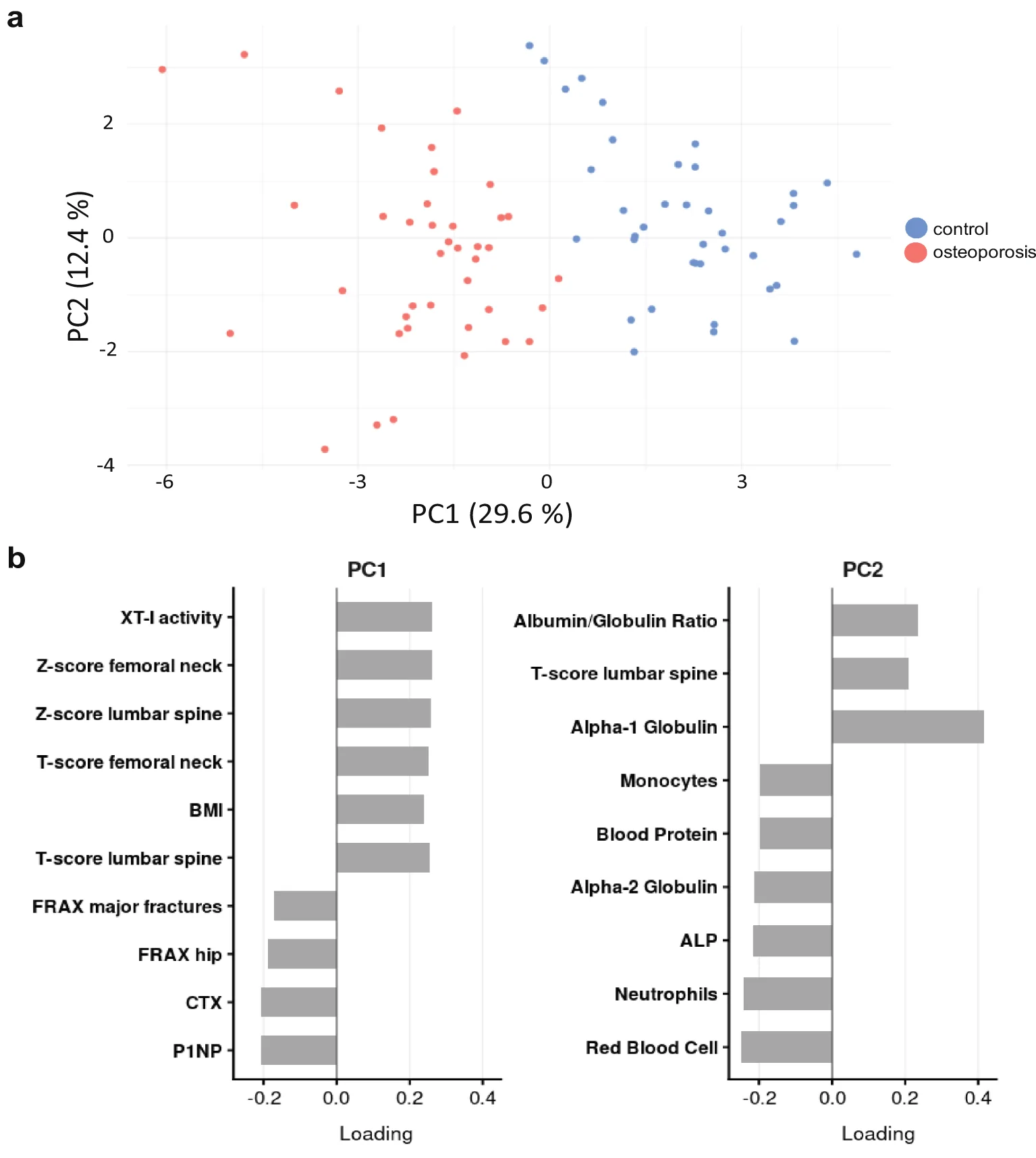
Principal component analysis and loading of all parameters activity measured in osteoporosis patients and controls. Serum levels were compared between postmenopausal osteoporosis patients (*n* = 40) and age-matched healthy controls (*n* = 40). (**a**) Score plot of the first two principal components.Each dot represents one participant; osteoporosis patients are shown in red, controls in blue. (**b**) Variable loadings for PC1 and PC2, displaying the ten parameters with the highest absolute loading values for each component. Positive loadings indicate a contribution in the direction of increasing PC score; negative loadings indicate an inverse contribution.

Principal component analysis was applied to all parameters measured to examine the multivariate structure of the dataset. PC1 accounted for 29.6% and PC2 for 12.4% of the total variance (Fig. 5a). Osteoporosis patients clustered predominantly at negative PC1 values in the score plot, whereas controls were located toward positive PC1 values, with a partial overlap in the central region reflecting the continuous nature of group separation in multidimensional space. Variable loading analysis (Fig. 5b) revealed that PC1 was primarily driven by bone density–related parameters, including T-scores and Z-scores at the femoral neck and lumbar spine, as well as XT-I activity, all contributing positively. By contrast, FRAX hip, FRAX major fractures, CTX, and P1NP loaded negatively on PC1. This pattern identifies PC1 as the principal axis of osteoporotic disease severity, contrasting lower bone density and XT-I activity with increased fracture risk and bone turnover. The XT-I activity clustered together with bone density parameters on the positive side of PC1, supporting its association with a more favorable skeletal phenotype. The XT-II activity, by contrast, did not show a relevant contribution to PC1, further distinguishing it from XT-I regarding disease-related variance. PC2 was characterized by positive loadings of proteins related to serum composition, including alpha-1 globulin and the albumin/globulin ratio, alongside contributions from the lumbar spine T-score. Negative loadings were observed for age, neutrophils, red blood cells, and additional protein-related parameters. This suggests that PC2 captures a secondary axis reflecting systemic or hematological variation rather than core osteoporotic disease processes. Overall, the PCA demonstrates that XT-I activity aligns with BMD and opposes fracture risk and turnover markers along the primary axis of variation, supporting its relevance as a component of the osteoporotic phenotype.

## Discussion

We demonstrate in this study, for the first time, that serum XT-I activity is significantly reduced in postmenopausal women with osteoporosis compared to age- and gender-matched healthy controls. Due to an increasingly ageing population, osteoporosis is one of the most prevalent diseases worldwide, leading to substantial morbidity and mortality, particularly among women^26^. Postmenopausal women are especially disproportionately affected due to the sharp decline in estrogen levels disrupting the physiological balance of bone remodeling^4^. The molecular mechanisms of osteoporosis are due to the increased activity of osteoclasts, decreased activity of osteoblasts, or both, which leads to an imbalance in the bone remodeling process with accelerated bone resorption and attenuated bone formation^7^. The reduction in XT-I activity observed was isoform-specific, as XT-II activity did not differ significantly between groups. This finding was enabled by a recently developed SPE-UPLC-MS/MS-based assay that allows the selective, simultaneous quantification of XT-I and XT-II activity from a single serum sample^27^. Prior studies investigating circulating XT activity in other disease contexts were based on an assay lacking strong isoenzyme selectivity, precluding a direct comparison of isoform-specific contributions to disease-related ECM remodeling^23,24^. The present study is, therefore, the first to apply isoform-specific XT activity measurement in an osteoporosis cohort. Furthermore, the divergent behavior of XT-I and XT-II highlights the added value of isoform-specific analysis. Although XT-II activity was not significantly altered in postmenopausal women, weak associations with bone-related parameters were noted, including a moderate positive correlation with the T-score at the lumbar spine and negative correlations with FRAX-derived fracture risk parameters. These effects were smaller than those observed for XT-I, nonetheless, they suggest that XT-II may reflect aspects of postmenopausal bone-related homeostasis as well. Notably, XT-II represents the predominant XT isoform in human serum, whereas XT-I appears to be more dynamically regulated in responsive to pathological alterations. These findings suggest that XT-I and XT-II may capture different aspects in bone biology. The XT-I activity consistently covaried with BMD parameters and inversely with fracture risk scores and bone turnover markers across multiple analytical approaches, including pairwise correlation analysis, a focused FDR-corrected association analysis, and PCA. Thus, XT-I was identified as a novel serum parameter associated with the osteoporotic phenotype, with potential relevance as both a reflector of ECM remodeling and a disease-specific biomarker. The reduction in circulating XT-I activity observed in osteoporosis patients is mechanistically plausible. The XT-I catalyzes the rate-limiting first step of GAG chain biosynthesis by transferring xylose to defined serine residues of PG core proteins^10^. The GAGs are integral constituents of the bone ECM and exert regulatory functions in bone remodeling, including direct modulation of RANKL–RANK interactions and control over the bioavailability of bone-anabolic growth factors^20,21^. Reduced XT-I activity may therefore reflect impaired PG biosynthesis in osteoporotic bone, leading to quantitative or qualitative deficiencies in the GAG composition of the bone matrix. The inhibitory role of GAGs and PG in osteoclastogenesis is well established and operates through direct and indirect mechanisms. Direct GAG-mediated inhibition of osteoclast differentiation has been demonstrated in vitro, where treatment of the RAW264 osteoclast cell line with chondroitin sulfate E suppressed osteoclastogenesis via blockade of osteoactivin and α5β3 integrin^28^. Indirect modulation occurs through altered osteoblast-osteoclast communication: hyaluronic acid treatment of bone marrow-derived osteoblasts reduced RANKL secretion, thereby attenuating downstream osteoclastogenesis^29^. Götting et al. demonstrated that elevated XT activity in the serum of systemic sclerosis patients correlated with increased hyaluronic acid levels^30^, suggesting that reduced XT-I activity in osteoporotic bone may be associated with diminished hyaluronic acid expression in osteoblasts, which could further impair the osteoblast-mediated suppression of osteoclastogenesis. Consequently, reduced GAG availability resulting from decreased XT-I activity could weaken inhibitory control points in osteoclast differentiation, thereby promoting increased bone resorption characteristic of postmenopausal osteoporosis. Circulating XT-I activity may therefore serve as a systemic indicator of impaired matrix synthesis capacity, reflecting altered PG turnover.

The strong positive correlations between XT-I activity and BMD at both the femoral neck and lumbar spine underscore the relationship between XT-I activity and skeletal integrity. Notably, these associations held for both T-scores, which reflect deviation from the peak bone mass, and Z-scores, which account for age-related bone loss. The consistency across measurement sites and score types argue against a measurement-specific artefact and supports a genuine biological link between XT-I activity and bone quality. The inverse associations of XT-I activity with CTX and P1NP are of particular interest. The effect sizes observed are comparable to those reported for established bone turnover markers from other studies^31,32^, indicating that XT- I may provide complementary information relative to those bone turnover markers. Elevated CTX and P1NP levels indicate accelerated bone remodeling, a hallmark of postmenopausal bone loss^33^, yet, their negative correlation with XT-I suggests that increased remodeling activity is not accompanied by adequate ECM replenishment. This functional decoupling of elevated bone turnover in the context of reduced PG biosynthetic capacity, as reflected by diminished XT-I activity, may contribute to the progressive deterioration of bone microarchitecture beyond what is captured by BMD alone. The additional negative correlation with FRAX scores is clinically meaningful, linking XT-I to not only bone density but also to fracture risk, which is the primary clinical endpoint in osteoporosis^34^. It should be noted that FRAX is a composite score derived from several established clinical risk factors, including age, BMI and femoral neck BMD^34,35^. Consequently, correlations between FRAX and these individual parameters are not independent but are partly inherent to the mathematical structure of the FRAX algorithm. Therefore, the observed negative associations between XT-I activity and FRAX scores should not be interpreted as independent biological relationships. Rather, they reflect the association of XT-I activity with established clinical determinants of fracture risk. Importantly, XT-I activity remained significantly associated with individual measures of skeletal integrity, including BMI and BMD, supporting its potential value as a biomarker reflecting ECM remodeling in postmenopausal osteoporosis.

Moderate positive associations were also observed between XT-I activity and BMI as well as age at menopause. The association with BMI may partly reflect the well-established protective effect of higher body weight on BMD^36,37^, as increased mechanical loading stimulates osteoblastic activity and bone formation^38^, potentially promoting ECM and PG synthesis in bone. Furthermore, the association with age at menopause is biologically coherent, as a later onset of menopause implies a longer duration of endogenous estrogen exposure, which is protective for bone density^39,40^. Consistent with this, postmenopausal women with osteoporosis have been reported to exhibit significantly lower circulating estradiol concentrations than healthy postmenopausal controls, and serum estradiol levels positively correlate with BMD^41^. Estrogen has been shown to regulate ECM metabolism and modulate transforming growth factor-β signaling, a key pathway involved in XT-I regulation^42^. Although none of the women included in the present study received hormone replacement therapy, circulating estradiol concentrations and other sex hormone measurements were not measured. Therefore, a potential relationship between estrogen deficiency and reduced XT-I activity could not be directly evaluated in the present study and should be investigated in future prospective studies incorporating endocrine profiling.

Importantly, XT-I activity showed no significant correlation with chronological age, indicating that the observed associations are not confounded by general aging effects and are specific to osteoporosis-related biological changes. In support of this, Kleine et al. reported that XT-I activity tends to increase with age in healthy blood donors, with a significant elevation observed in individuals over 40 years old compared to younger subjects^27^. This further suggests that the reduction of XT-I activity in osteoporosis is not a simple consequence of aging but reflects disease-specific alterations.

The PCA provided an integrative view of the multivariate structure of the dataset, which in this study, included not only established bone parameters but also a panel of laboratory parameters. PC1emerged as a continuum of osteoporotic disease severity: positive loadings for bone density parameters and XT-I activity contrasted with negative loadings for bone resorption markers and FRAX scores, with osteoporosis patients clustering toward negative and controls toward positive PC1 values. The positioning of XT-I activity on PC1, in close alignment with T-scores and Z-scores and in opposition to CTX, P1NP, and fracture risk scores, suggests that XT-I is not an isolated biomarker but an integral component of the biological landscape that distinguishes osteoporotic from healthy bone. PC2 was driven by hematological and serum protein parameters, including albumin/globulin ratio, alpha-1 and alpha-2 globulin fractions, monocytes, neutrophils, and red blood cell count, alongside ALP^43–45^. This component probably reflects a systemic variation in inflammatory status independent of the osteoporotic disease. Crucially, XT-I activity showed no relevant contribution to PC2, confirming its specificity for the primary skeletal disease axis. The biological relevance of XT-I in skeletal biology is further supported by recent experimental studies demonstrating that *XYLT1* deficiency in human mesenchymal stem cells alters osteogenic differentiation and ECM composition. These findings suggest that XT-I contributes to bone matrix homeostasis beyond its established role during skeletal development and support the concept that altered XT-I activity may influence bone remodeling through changes in PG biosynthesis and ECM organization^15^. This is further supported by rare genetic disorders: Pathogenic mutations in *XYLT1* cause DBQD2, with prominent skeletal abnormalities but not osteoporosis, while pathogenic mutations in *XYLT2* cause SOS, which features severe osteoporosis as a central manifestation^16,17^. Both conditions demonstrate that impaired XT function compromises skeletal integrity directly. However, this study does not suggest that osteoporosis is primarily caused by XT-I deficiency. Rather, the reduction in circulating XT-I activity observed in postmenopausal osteoporosis probably reflects diminished ECM biosynthesis secondary to estrogen deficiency. This interpretation is consistent with the central role of estrogen in regulating bone turnover, regulating PG synthesis and modulating transforming growth factor-β signaling. Since XT-II is not regulated by transforming growth factor-β, estrogen deficiency would be expected to selectively impair XT-I activity without affecting XT-II, which is consistent with the isoform-specific pattern observed in the present study. The occurrence of severe osteoporosis in SOS highlights a fundamentally different and yet not understood mechanism. Perhaps XT-I and XT-II regulate distinct aspects of bone ECM biology. It is suggested that quantitatively reduced XT-I activity may similarly contribute to bone fragility in the more common context of postmenopausal osteoporosis, extending the pathophysiological concept of XT deficiency from monogenic disorders to an acquired, multifactorial disease. Finally, from a clinical perspective, XT-I activity offers information complementary to that of established bone turnover markers. Both CTX and P1NP reflect the rate of bone resorption and formation respectively, but do not directly capture the status of the bone ECM. XT-I, by contrast, provides an insight into the matrix synthesis capacity that determines whether bone formed during remodeling is structurally competent. The LC-MS/MS-based enzyme activity assay used here is technically feasible for clinical laboratory adaptation, representing a realistic pathway toward clinical implementation^27^. However, before XT-I can be considered as a clinical tool, its diagnostic performance, reference intervals, and biological variability need to be established in larger, prospective cohorts. The cohort comprised exclusively postmenopausal women, limiting the applicability to men or premenopausal women. The cross-sectional design precludes conclusions regarding causality or the temporal dynamics of XT-I activity during disease progression. Furthermore, direct mechanistic evidence linking circulating XT-I activity to bone-specific GAG composition is lacking and should be addressed through future longitudinal studies and bone ECM analyses. In conclusion, this study identifies serum XT-I activity as a novel, isoform-specific parameter associated with BMD, fracture risk, and the multivariate structure of postmenopausal osteoporosis. Its consistent alignment with bone density parameters across correlation analysis and PCA, together with its inverse association with bone turnover and fracture risk markers and its independence from hematological and nonspecific systemic variation, suggests that XT-I reflects a fundamental aspect of ECM remodeling in osteoporotic bone. These findings provide a rationale for further exploration of XT-I as a biomarker and a contributor and putative therapeutic target to the pathophysiology of postmenopausal bone loss.

## Materials and methods

### Patients

The study included 40 postmenopausal women diagnosed with osteoporosis (mean age 64.03 ± 3.96 years) and 40 age-matched healthy postmenopausal controls (mean age 62.42 ± 3.10 years). All procedures were conducted in accordance with the Helsinki Declaration and received approval from the local ethics committee (Internal Review Board, University of L’Aquila, formerly “Academic Ethics Committee,” D.R. No. 206/2013, amended by D.R. No. 46/2017)^46^. Written informed consent was obtained from every participant prior to study inclusion. BMD was measured at the hip and lumbar spine using dual-energy X-ray absorptiometry (DXA) with a Hologic QDR 4500 W device. Results were reported as T-scores, representing the number of standard deviations by which an individual’s BMD differs from the mean BMD of a healthy reference population. The BMD was assessed at the femoral neck and lumbar spine and reported as both T- and Z-scores. Osteoporosis was diagnosed following the criteria established by the World Health Organization^47^: a T-score below − 2.5 indicates osteoporosis, values between − 1.0 and − 2.5 reflect osteopenia, and a T-score above − 1.0 is considered within the normal range. In addition, the Z-score was calculated: this provides a comparison with the bone density of people of the same age and gender as the patient. A negative Z-score of − 2.5 or lower should raise the suspicion of a secondary cause of osteoporosis^48^. Each participant underwent a thorough physical examination and a comprehensive medical history assessment, alongside the measurement of anthropometric parameters including body weight, height, and body mass index (BMI). Imaging studies, including spinal radiography and vertebral morphometry were performed to identify potential asymptomatic osteoporotic fractures. Ten-year fracture probabilities for major osteoporotic and hip fractures were estimated for all participants using the Fracture Risk Assessment Tool (FRAX)^35^. Complete blood count and serum electrophoresis were obtained from all participants to exclude hematological disorders and assess the general health status. Routine laboratory parameters reflecting hepatic, renal, and metabolic function were recorded, including serum calcium (Ca²⁺), vitamin D (1α,25- dihydroxyvitamin D₃), parathyroid hormone (PTH), and alkaline phosphatase (ALP). Bone turnover was further characterized in both osteoporosis patients and controls by measuring the bone resorption marker C-terminal telopeptide of type I collagen (CTX) and the bone formation markers N-terminal propeptide of type I procollagen (P1NP), bone alkaline phosphatase (Ostase) and osteocalcin, serving collectively as indirect indicators of osteoclast and osteoblast activity.

The control group was recruited as an age-adjusted clinically healthy convenience sample and was not intended to represent a population-based reference cohort. Controls fulfilled the inclusion criterion of a T-score > − 1.0 at both the femoral neck and lumbar spine, showed no evidence of osteoporosis. None of the participants took medications known to affect bone metabolism or had conditions associated with secondary osteoporosis^49^. A comprehensive summary of all important bone-related clinical and laboratory parameters for both groups is presented in Table 1 and Table S1.

**Table 1:** Clinical, laboratory and anthropometric bone-related findings (means ± SD) of osteoporosis patients and controls.

Bone-related parameters	Controls [*n* = 40]	Osteoporosis [*n* = 40]
Age [years]	62.42 ± 3.10	64.03 ± 3.96
Menopause [years]*	51.50 ± 7.49	48.97 ± 7.47
Years since Menopause [years]*	10.93 ± 8.05	15.05 ± 7.63
BMI [kg/m^2^]*	28.83 ± 1.04	24.58 ± 0.76
BMD [T-score femoral neck]*	0.05 ± 0.87	-2.20 ± 0.66
BMD [T-score lumbar spine]*	-0.15 ± 0.74	-3.15 ± 0.69
BMD [Z-score femoral neck]*	0.86 ± 0.79	-0.92 ± 0.99
BMD [Z-score lumbar spine]*	1.45 ± 0.38	-1.43 ± 0.40
CTX [pg/mL]*	360.77 ± 15.51	474.75 ± 16.58
P1NP [ng/mL]*	49.55 ± 2.42	61.88 ± 2.22
FRAX [major fractures]*	5.32 ± 2.18	10.83 ± 5.44
FRAX [hip]*	0.55 ± 0.91	3.38 ± 3.02
ALP [U/L]	71.10 ± 0.96	78.93 ± 0.83
PTH [pg/mL]	47.95 ± 3.18	45.31 ± 3.31
Vitamin D [ng/mL]	31.50 ± 6.85	35.93 ± 6.41
Ca^2+^ [mg/dL]	9.34 ± 0.44	9.58 ± 0.40
Ostase [µg/L]	12.50 ± 5.16	14.10 ± 6.33
Osteocalcin [ng/mL]	24.19 ± 1.26	26.24 ± 2.09

*Statistically significant intergroup difference between osteoporosis patients and controls (*p* ≤ 0.05, Mann-Whitney U test).

### Mass spectrometric XT activity assay

Intracellular XT-I and XT-II enzyme activity was quantified from sera using a mass spectrometry-based enzymatic assay, adapting a methodology described previously. This approach measured relative XT-I and XT-II activity by monitoring the enzyme-mediated incorporation of xylose into specific acceptor peptides over a predetermined timeframe. Each reaction contained XT-I or XT-II-specific acceptor peptides (AP1 or AP2, 10 µL at 60 µg/mL), UDP-xylose substrate (10 µL at 80 µg/mL), Mg/Mn cofactor solution (2.5 µL at 5 mmol/L), MES buffer (2.5 µL at 25 mmol/L, pH 6.5), and 25 µL of the corresponding serum sample. The mixtures were incubated for 2 h at 37 °C, with assays conducted simultaneously for both AP1 and AP2 acceptor peptides. Enzymatic reactions were stopped by thermal denaturation (99 °C for 10 min), followed by centrifugation (10,000 × g for 10 min at 4 °C). The resulting supernatants (20 µL from each AP1 and AP2 reaction) were pooled and diluted with 60 µL of water before analytical processing. Detection and quantification of the xylosylated peptide products (AP1-Xyl and AP2-Xyl) were achieved through solid-phase extraction coupled with ultra-performance liquid chromatography tandem mass spectrometry (SPE-UPLC-MS/MS) using a single multiple reaction monitoring (MRM) protocol. Chromatographic separation and mass spectrometric analysis followed established procedures. Electrospray ionization operated in positive mode with the following parameters: capillary voltage 2.0 kV, desolvation temperature 600 °C, desolvation gas flow 900 L/h, and collision gas flow 0.15 mL/min. Each transition was monitored with a 0.1 s dwell time. Enzyme activity levels were determined through MRM peak area integration, with data collection and automated processing conducted using TargetLynx software (version 4.2 SCN997), integrated within the MassLynx Mass Spectrometry Software platform (Waters, Massachusetts, USA; available at: https://www.waters.com/nextgen/nl/en/products/informatics-and-software/mass-spectrometry-software/masslynx-mass-spectrometry-software.html).

### Statistical analysis

All statistical analyses were performed using GraphPad Prism (version 10.0; GraphPad Software LLC, San Diego, CA, USA; available at https://www.graphpad.com/) and R (version 4.3.3; R Foundation for Statistical Computing, Vienna, Austria; available at: https://www.r-project.org/). The non-parametric two-tailed Mann–Whitney U test was applied for intergroup comparisons. Pairwise Pearson correlation coefficients (*r*) were calculated to assess linear associations between all continuous variables and visualized as a colour-coded correlation matrix. Individual pairwise Pearson correlations were computed for all measured parameters and p-values were adjusted for multiple comparisons using the Benjamini–Hochberg false discovery rate (FDR) method; FDR-adjusted p-values are reported throughout and visualized as a ranked dot plot. Principal component analysis (PCA) was conducted on Z-score-standardized data to investigate the multivariate structure of the dataset and the contribution of XT activity to disease-related variance. The ten variables with the highest absolute loading values for PC1 and PC2 are displayed. Data are presented as mean ± standard deviation (SD). Differences with a p-value ≤ 0.05 were considered statistically significant.

## Supplementary Information

Below is the link to the electronic supplementary material.

Supplementary Material 1

## Data Availability

The datasets used and/or analyzed during the current study are available from the corresponding author upon reasonable request.
