# Supplementary material for: Xylosyltransferase-I serum activity is a promising biomarker for postmenopausal osteoporosis
Source: Sci Rep. 2026 Aug 20;16:26204. doi: 10.1038/s41598-026-66889-0 (PMC13493843; doi:10.1038/s41598-026-66889-0)
Supplement: Supplementary file 1 [file 41598_2026_66889_MOESM1_ESM.pdf]

Supplementary Information

Table S1: Clinical, laboratory and anthropometric findings (means ± SD) of osteoporosis patients and controls.

Laboratory-related parameters	Controls [n = 40]	Osteoporosis [n = 40]
Red Blood Cell [x10^6/µL]	6.04 ± 1.26	6.13 ± 2.09
Hemoglobin [g/dL]	13.15 ± 1.08	13.43 ± 1.04
White Blood Cell [x10^6/µL]	4.53 ± 0.44	4.62 ± 0.40
Platelets [x10^3/µL]	270.80 ± 65.42	258.00 ± 60.82
Neutrophils [x10^3/µL]	3.40 ± 0.98	3.62 ± 1.93
Leucocytes [x10^3/µL]	2.01 ± 0.50	1.83 ± 0.61
Monocytes [x10^3/µL]	0.34 ± 0.08	0.36 ± 0.12
Eosinophiles [x10^3/µL]	0.15 ± 0.09	0.16 ± 0.11
Basophiles [x10^3/µL]	0.04 ± 0.01	0.05 ± 0.04
CRP [mg/dL]	3.03 ± 3.70	3.72 ± 10.75
Creatinine [mg/dL]*	0.79 ± 0.17	0.71 ± 0.11
ALT [U/L]	19.27 ± 5.83	22.57 ± 11.01
AST[U/L]	20.43 ± 8.74	18.52 ± 8.17
GGT[U/L]	26.35 ± 29.23	21.45 ± 11.88
Blood Protein [g/dL]	7.43 ± 0.48	7.54 ± 0.41
TSH[µUI/mL]	1.77 ± 1.14	1.38 ± 0.81
fT_3 [pg/mL]	3.28 ± 0.96	3.36 ± 0.83
fT_4 [pg/mL]	1.29 ± 0.19	1.28 ± 0.20
Blood Protein [g/dL]	7.43 ± 0.48	7.54 ± 0.41
Albumin [g/dL]	4.45 ± 0.47	4.55 ± 0.27
IgG [g/dL]	1.06 ± 0.26	1.03 ± 0.26
Alpha-1 Globulin [g/dL]	0.40 ± 0.65	0.30 ± 0.07
Alpha-2 Globulin [g/dL]	0.73 ± 0.14	0.73 ± 0.13
Beta-1 Globulin [g/dL]	0.45 ± 0.07	0.44 ± 0.08
Beta-2 Globulin [g/dL]	0.38 ± 0.08	0.39 ± 0.08
Albumin/Globulin Ratio [%]	1.73 ± 1.37	1.58 ± 0.22

Legend: C-reactive protein (CRP), Aspartate Aminotransferase (ALT), Alanine Aminotransferase (AST), Gamma Glutamyltransferase (GGT), Thyroid-stimulating hormone (TSH), free triiodothyronine (fT), Immunoglobulin G (IgG); *Statistically significant difference between osteoporosis patients and controls ($p \leq 0.05$, Mann-Whitney U test).
